# Evidence for a personalized early start of norepinephrine in septic shock

**DOI:** 10.1186/s13054-023-04593-5

**Published:** 2023-08-22

**Authors:** Xavier Monnet, Christopher Lai, Gustavo Ospina-Tascon, Daniel De Backer

**Affiliations:** 1 AP-HP, Service de Médecine Intensive-Réanimation, Hôpital de Bicêtre, DMU 4 CORREVE, Inserm UMR S_999, FHU SEPSIS, CARMAS, Université Paris-Saclay, 78 rue du Général Leclerc, 94270 Le Kremlin-Bicêtre, France; 2 Department of Intensive Care Medicine, Fundación Valle del Lili, Av. Simón Bolívar Cra. 98, Cali, Colombia; 3 Translational Research Laboratory in Critical Care Medicine (TransLab-CCM), Universidad ICESI, Cali, Colombia; 4 Department of Intensive Care, CHIREC Hospitals, Université Libre de Bruxelles, Brussels, Belgium

**Keywords:** Fluids, Fluid accumulation, Catecholamine, Systemic venous return, Vasodilatation

## Abstract

During septic shock, vasopressor infusion is usually started only after having corrected the hypovolaemic component of circulatory failure, even in the most severe patients. However, earlier administration of norepinephrine, simultaneously with fluid resuscitation, should be considered in some cases. Duration and depth of hypotension strongly worsen outcomes in septic shock patients. However, the response of arterial pressure to volume expansion is inconstant, delayed, and transitory. In the case of profound, life-threatening hypotension, relying only on fluids to restore blood pressure may unduly prolong hypotension and organ hypoperfusion. Conversely, norepinephrine rapidly increases and better stabilizes arterial pressure. By binding venous adrenergic receptors, it transforms part of the unstressed blood volume into stressed blood volume. It increases the mean systemic filling pressure and increases the fluid-induced increase in mean systemic filling pressure, as observed in septic shock patients. This may improve end-organ perfusion, as shown by some animal studies. Two observational studies comparing early *vs.* later administration of norepinephrine in septic shock patients using a propensity score showed that early administration reduced the administered fluid volume and day-28 mortality. Conversely, in another propensity score-based study, norepinephrine administration within the first hour following shock diagnosis increased day-28 mortality. The only randomized controlled study that compared the early administration of norepinephrine alone to a placebo showed that the early continuous administration of norepinephrine at a fixed dose of 0.05 µg/kg/min, with norepinephrine added in open label, showed that shock control was achieved more often than in the placebo group. The choice of starting norepinephrine administration early should be adapted to the patient’s condition. Logically, it should first be addressed to patients with profound hypotension, when the arterial tone is very low, as suggested by a low diastolic blood pressure (e.g. ≤ 40 mmHg), or by a high diastolic shock index (heart rate/diastolic blood pressure) (e.g. ≥ 3). Early administration of norepinephrine should also be considered in patients in whom fluid accumulation is likely to occur or in whom fluid accumulation would be particularly deleterious (in case of acute respiratory distress syndrome or intra-abdominal hypertension for example).

**Supplementary Information:**

The online version contains supplementary material available at 10.1186/s13054-023-04593-5.

## Background

During septic shock, vasopressors, first and foremost norepinephrine, are administered to restore tissue perfusion pressure. For a long time, the rule was to start vasopressors only after correcting the hypovolaemic component of circulatory failure, even in most severe patients. However, starting norepinephrine earlier, simultaneously with fluid resuscitation, should be considered. Initiating infusion peripherally may avoid delays in norepinephrine administration, as recognized by the Surviving Sepsis Campaign guidelines. As this attitude is safe when low doses are used [1], the real question is: what may be the advantages of early start of norepinephrine?

## Only early vasopressor administration can quickly correct severe hypotension

Duration and depth of hypotension are strongly associated with poor outcomes in septic shock. Volume expansion may induce variable responses in arterial pressure, first because its effects on stroke volume are inconstant, delayed, and transitory, second because arterial elastance, which determines the relationship between the fluid-induced changes in flow and in arterial pressure, is variable. In the case of profound, life-threatening hypotension, relying only on fluids to restore blood pressure may unduly prolong hypotension and consequently organ hypoperfusion. Conversely, norepinephrine rapidly increases arterial pressure, its dose being adjusted according to the arterial pressure target (Additional file 1: Fig. S1).

## Fluids and norepinephrine increase cardiac output synergistically

By binding venous adrenergic receptors, norepinephrine transforms part of the unstressed blood volume into stressed blood volume. It thus increases the mean systemic filling pressure (Pmsf), the upstream pressure of systemic venous return. This fluid-like effect has been demonstrated in septic shock patients [2] or after cardiac surgery [3]. This increase in Pmsf leads to a significant increase in cardiac preload [4, 5] which, in the event of preload responsiveness, significantly increases cardiac output [2].

Once started, norepinephrine may even increase the effectiveness of future fluid loadings. Once the venous reservoir is constricted by norepinephrine, the fluid administered increases the stressed blood volume more than if it spreads in a dilated venous system. In septic shock patients, the effect on Pmsf of a passive leg raising, mimicking the effects of a fluid challenge, was greater at a higher dose than at a lower dose of norepinephrine [6]. These synergistic effects of norepinephrine and fluids could decrease the total fluid volume required for initial resuscitation of septic shock, which is associated with increased mortality.

To this preload-related synergy between norepinephrine and fluid, an improvement in cardiac output may be added through an increased cardiac contractility [7], possibly related to beta-adrenergic stimulation or improved coronary perfusion [8].

The final question is of course whether early norepinephrine improves tissue perfusion and/or organ function. Only experimental data are available. In two studies in animals with endotoxic shock, early norepinephrine administration added to fluid infusion improved cardiac output, increased gut microvascular blood flow, and blunted the increase in lactate levels [5, 9].

## Early administration of norepinephrine during septic shock may improve clinical outcomes

Among the observational studies comparing early *vs.* later administration of norepinephrine, three used propensity scores. Ospina-Tascon et al. [10] and Xu et al. [11] showed that early administration of norepinephrine (< 1 h and < 3 h after shock diagnosis, respectively) reduced the volume of fluid administered and day-28 mortality.

Conversely, in another propensity score-based study, norepinephrine administration within the first hour following shock diagnosis increased day-28 mortality [12]. The main difference with the study by Ospina-Tascon et al. [10] is that, in the latter, tissue perfusion and preload responsiveness were assessed.

So far, only one randomized controlled study has compared the early administration of norepinephrine alone to the concomitant administration of a placebo [13]. Permpikul et al. randomized 310 septic shock patients to continuous administration of early norepinephrine at a fixed dose of 0.05 µg/kg/min versus placebo. If blood pressure was not restored, open-label administration of norepinephrine was allowed in both groups. In the “early norepinephrine” group, the primary outcome, i.e. shock control in the first 6 h, was achieved more often than in the other group [13].

In the recent CLOVERS [14] study, patients were assigned to either a restrictive fluid strategy (prioritizing vasopressors and lower fluid volumes) or a liberal fluid strategy (prioritizing higher fluid volumes before use of vasopressors) for 24 h [14]. There was no difference in day-90 mortality. However, this study did not test the early administration of norepinephrine in isolation. One fifth of the patients received vasopressors at time of randomization, and this proportion increased only to 59% in the restrictive fluid therapy group and 37% in the liberal group. [14]. Hence, there is no definitive proof from randomized trials that early administration improves survival, but most data gathered so far at least suggest that this approach is safe.

## Timing of norepinephrine administration should be individualized

From the expected effects of the early administration of norepinephrine, it should first be used in patients with severe hypotension even though there is no consensual definition about the level. On the other hand, it should be used in patients with very low arterial tone, as suggested by low diastolic blood pressure (e.g. ≤ 40 mmHg), or a high diastolic shock index (heart rate/diastolic blood pressure) (e.g. ≥ 3). Computation of arterial elastance may identify patients who would necessitate norepinephrine to correct hypotension, but this approach requires cardiac output monitors which are not available at this very early stage of resuscitation.

**Fig. 1:**
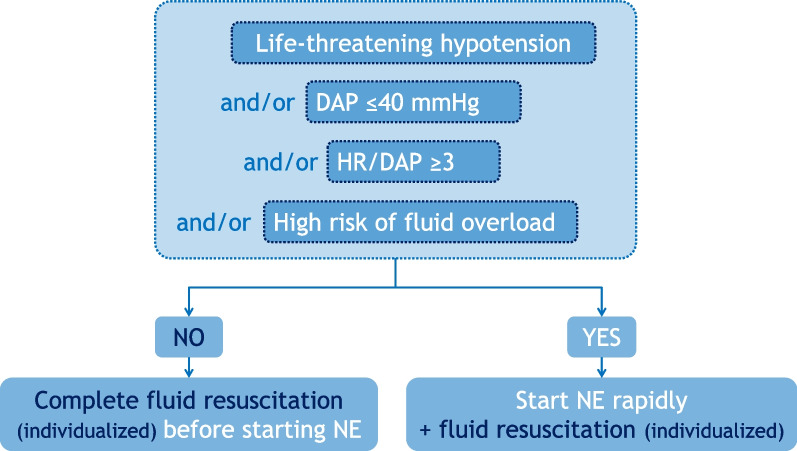
How to optimize timing of introduction of norepinephrine. Suggested flowchart for deciding when to introduce norepinephrine. DAP: Diastolic arterial pressure, HR/DAP: ratio between heart rate and DAP

Early administration of norepinephrine should also be considered in patients in whom fluid accumulation has occurred prior to hypotension, is likely to occur (patients who are anuric, who received large fluid amounts before hypotension occurred), or those in whom fluid accumulation would be particularly deleterious (e.g. in case of acute respiratory distress syndrome, left or right ventricular failure, or intra-abdominal hypertension) (Fig. 1). Administration of norepinephrine should go hand in hand with reasoned fluid administration, based on physiological needs and assessment of preload responsiveness, as in the study by Ospina Tascon et al. [10] (Fig. 1).

## Conclusions

Early administration of norepinephrine during shock may be justified in patients with profound vasoplegia and/or at high risk of fluid overload, along with a personalized fluid administration strategy [15].

### Supplementary Information

**Additional file 1: Figure S1** Potential benefits and risks of early administration of norepinephrine in septic shock

## Data Availability

Not applicable.

## Abbreviation

Pmsf: Mean systemic filling pressure
