# Supplementary material for: Evidence for a personalized early start of norepinephrine in septic shock
Source: Crit Care. 2023 Aug 22;27:322. doi: 10.1186/s13054-023-04593-5 (PMC10464210; doi:10.1186/s13054-023-04593-5)
Supplement: Supplementary file 1 — Additional file 1: Figure S1 Potential benefits and risks of early administration of norepinephrine in septic shock [file 13054_2023_4593_MOESM1_ESM.docx]

Evidence for a personalised early start of norepinephrine in septic shock

Xavier MONNET ^(1)^

Christopher LAI ^(1)^

Gustavo OSPINA-TASCON ^(2,3)^

Daniel DE BACKER ^(4)^

1. AP-HP, Service de médecine intensive-réanimation, Hôpital de Bicêtre, DMU 4 CORREVE, Inserm UMR S_999, FHU SEPSIS, CARMAS, Université Paris-Saclay, 78 rue du Général Leclerc, 94270, Le Kremlin-Bicêtre, France.
2. Department of Intensive Care Medicine, Fundación Valle del Lili, Av. Simón Bolívar Cra. 98, Cali, Colombia
3. Translational Research Laboratory in Critical Care Medicine (TransLab-CCM), Universidad ICESI, Cali, Colombia
4. Department of Intensive Care, CHIREC Hospitals, Université Libre de Bruxelles, Brussels, Belgium

*Address for correspondence*

Prof. Xavier MONNET

Service de médecine intensive-réanimation

Hôpital de Bicêtre

78, rue du Général Leclerc

94 270 Le Kremlin-Bicêtre

France

[xavier.monnet@aphp.fr](mailto:xavier.monnet@aphp.fr)

Supplemental figure 1

Potential benefits and risks of early administration of norepinephrine in septic shock
